# Supplementary material for: Automated optimization of force field parameters against ensemble-averaged measurements with Bayesian Inference of Conformational Populations
Source: ArXiv. 2026 Jun 23:arXiv:2402.11169v3. Preprint. [Version 3] (PMC13321333)
Supplement: Supplement 1 [file NIHPP2402.11169v3-supplement-1.pdf]

Supplemental Material

Automated optimization of force field parameters against ensemble-averaged measurements with Bayesian Inference of Conformational Populations

Robert M. Raddi and Vincent A. Voelz

Department of Chemistry, Temple University, Philadelphia, Pennsylvania 19122, USA

This PDF contains additional figures, tables, and computational details supporting the results presented in the main text.

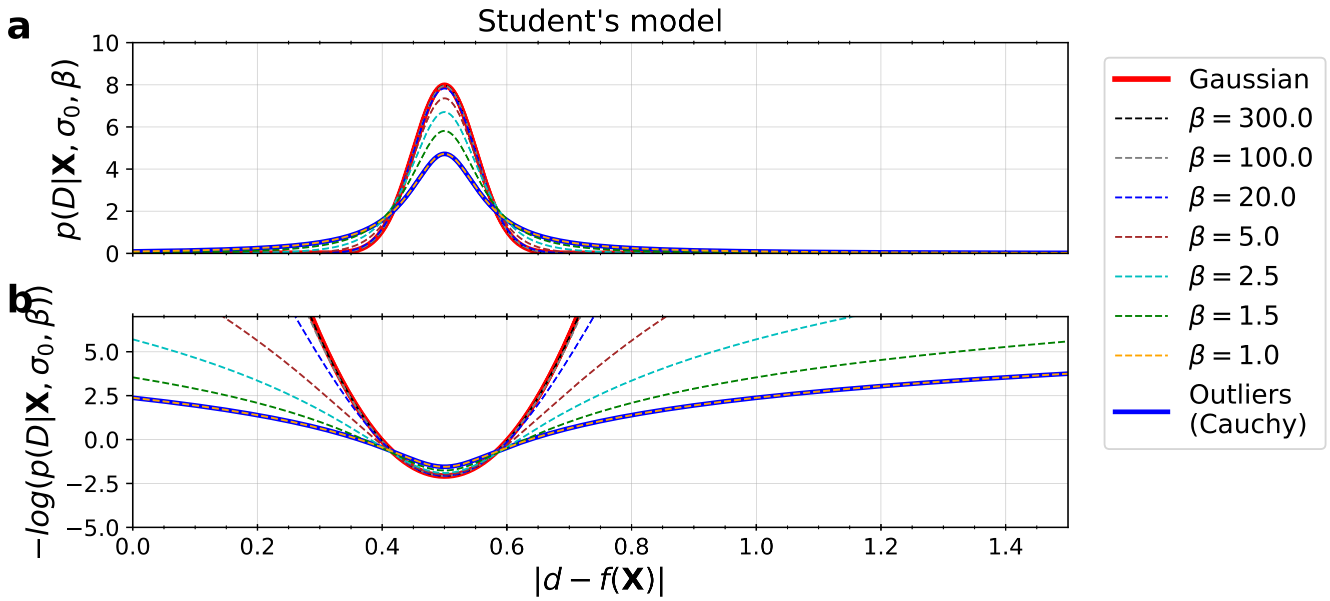

Figure S1. The probability density function (a) and energy landscape (b) of the marginal likelihood for the Student's model with respect to the replica-averaged forward model data $f(\mathbf{X})$. The colored curves are different values of nuisance parameter β. The Student's model is equivalent to the Outliers model (Cauchy) when $\beta = 1$, and as β goes to infinity, the pdf becomes Gaussian and the energy becomes harmonic.

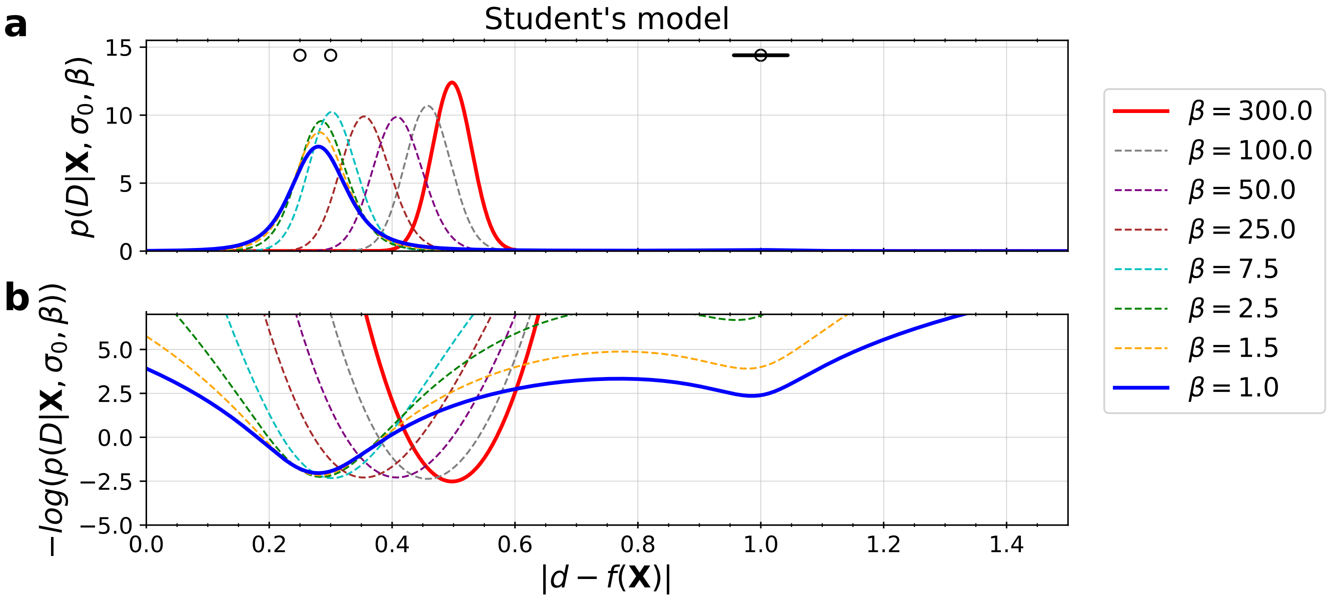

Figure S2. The probability density function (a) computed as $p(D|\mathbf{X},\sigma_0,\beta) = \prod_j^{N_j} p(d_j|\mathbf{X},\sigma_0,\beta)$ and energy landscape (b) of the marginal likelihood for the Student's model with respect to the replica-averaged forward model data $f(\mathbf{X})$ using multiple data points. Shown here, are three data points, two good data points $\{0.25, 0.3\}$ and one outlier $\{1.0\}$. The Cauchy likelihood ($\beta = 1$) is centered about the mean of the two good data points, demonstrating that this model can distinguish the good and bad data. The standard Gaussian likelihood ($\beta = 300$) is centered about the mean of all three data points. The colored curves are different values of nuisance parameter β.

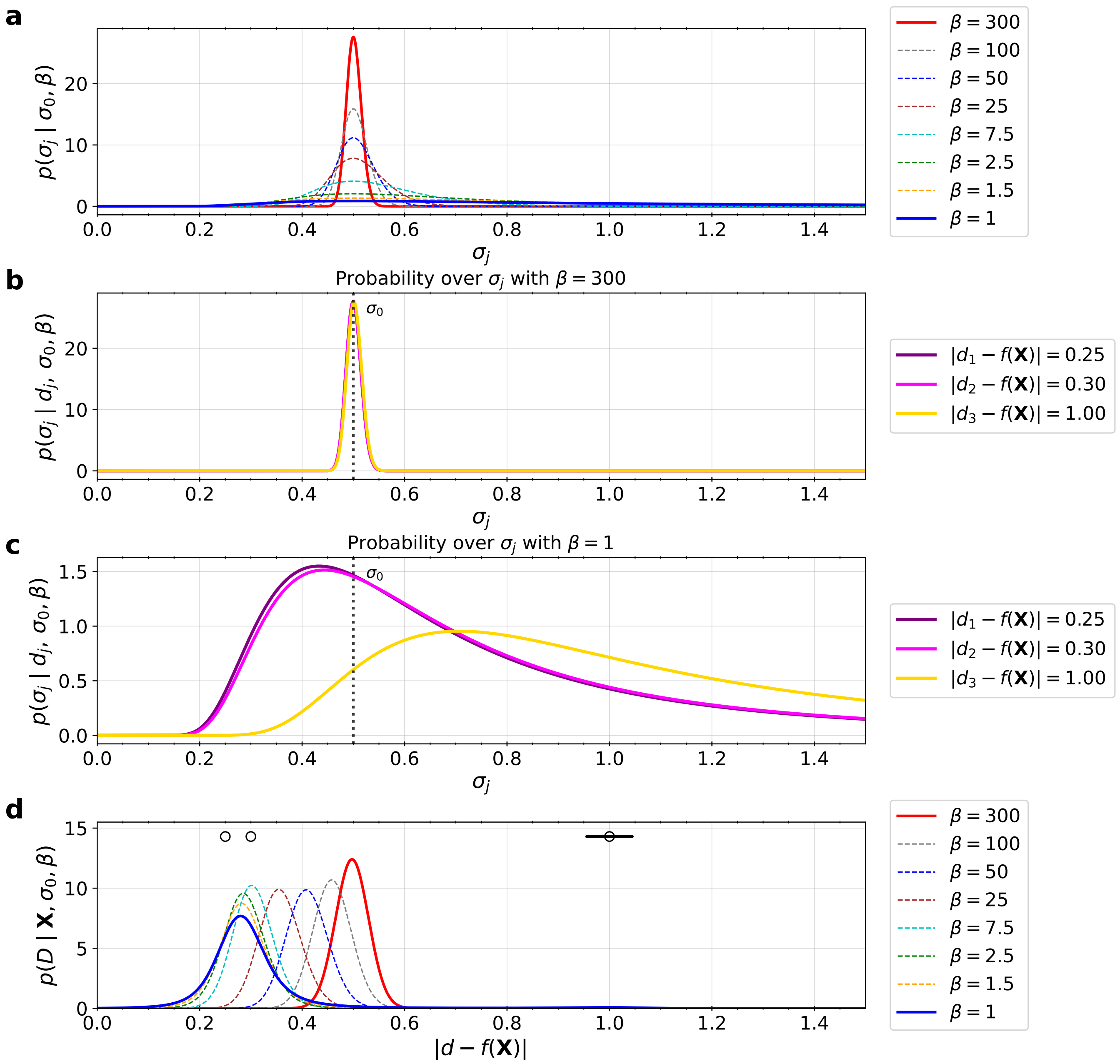

Figure S3. Hierarchical interpretation of the Student's likelihood model across the prior on uncertainties, latent uncertainty, and the marginalized likelihood. (a) Prior distribution over the latent uncertainties, $p(\sigma_j \mid \sigma_0, \beta)$, for a range of β values. The parameter β controls the tail behavior of the distribution: small β yields broader, heavy-tailed priors, while large β concentrates probability near $\sigma_j \approx \sigma_0$, approaching the Gaussian limit as $\beta \to \infty$. (b-c) Posterior distribution over σ_j for individual data points, $p(\sigma_j \mid d_j, \sigma_0, \beta) \propto p(d_j \mid \sigma_j)\, p(\sigma_j \mid \sigma_0, \beta)$, shown for two typical observations and one outlier using $\beta = 300$ and $\beta = 1$, respectively. The good data points ($|d_1 - f(\mathbf{X})| = 0.25$ and $|d_2 - f(\mathbf{X})| = 0.30$) favor values of σ_j near σ_0, whereas the outlier ($|d_3 - f(\mathbf{X})| = 1.0$) shifts probability toward larger σ_j, effectively inflating the local uncertainty. (d) Marginalized likelihood, $p(D \mid \mathbf{X}, \sigma_0, \beta) = \prod_j \int p(d_j \mid \sigma_j)\, p(\sigma_j \mid \sigma_0, \beta)\, d\sigma_j$, plotted as a function of the residual $|d - f(\mathbf{X})|$. The open circles mark the observed data (two good data points and one outlier). Decreasing β produces heavier tails in the likelihood, allowing the model to accommodate outliers without strongly penalizing deviations, while increasing β recovers a sharply peaked Gaussian-like likelihood.

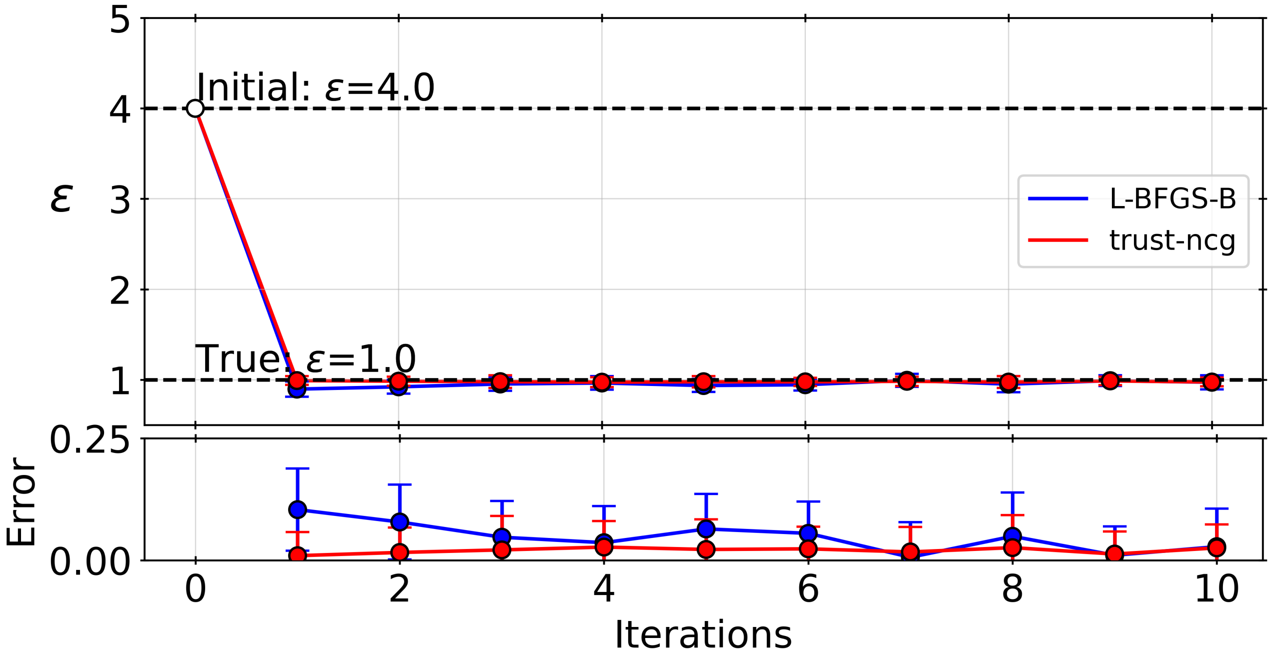

Figure S4. Trust-region optimization with BICePs is found to converge faster and be more robust than L-BFGS-B. Here, we compare first-order L-BFGS-B method (blue) to the second-order Trust-NCG optimization method (red). A total of 25 independent rounds of parameter (ε) optimizations using the BICePs score for a maximum of ten iterations. Data points are placed at the mean value for each iteration and error bars are the standard deviation. It is typical that the trust-ncg method reaches convergence slightly faster (the 4th iteration), and the L-BFGS-B method tends to slightly overshoot the "True" ε value on the first iteration.

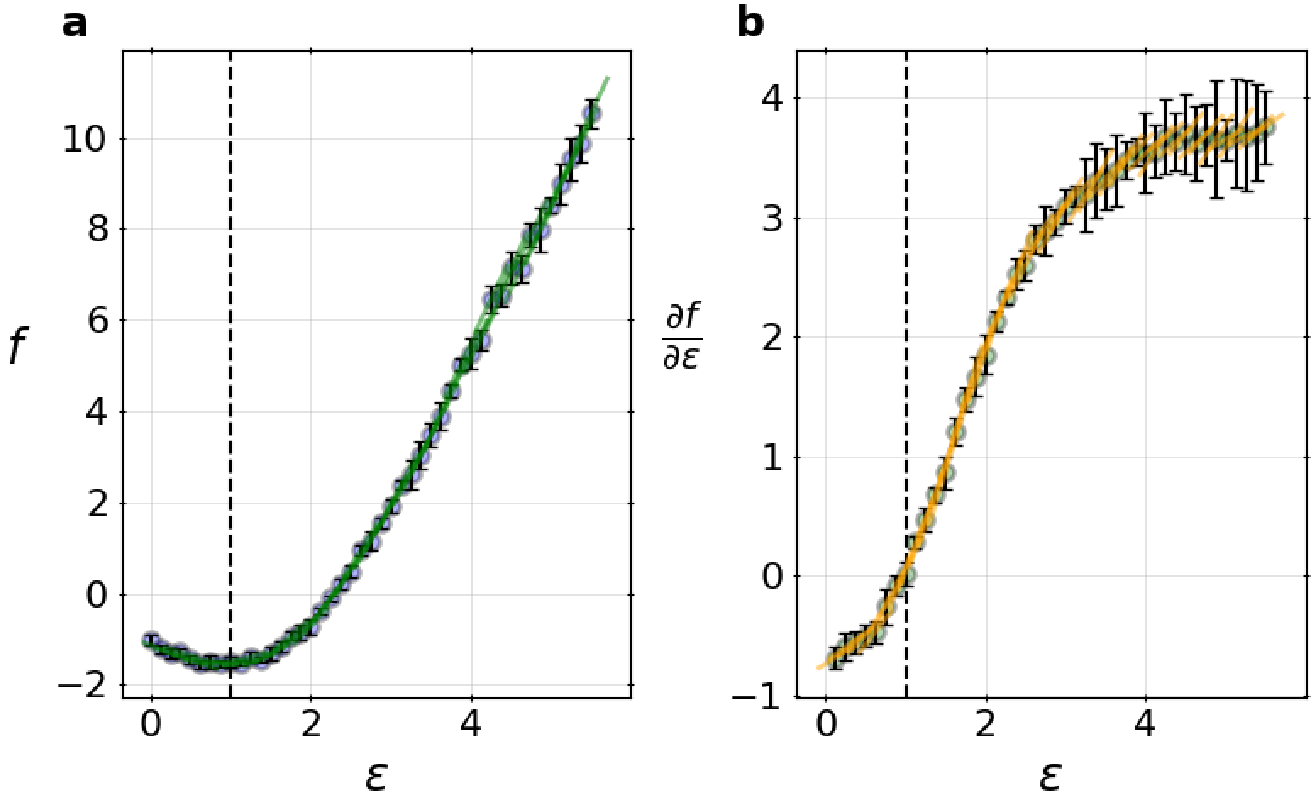

Figure S5. (a) 1-D scans over ε to reveal the landscape of the BICePs score (scatter dots). The green tangent lines are the derivatives at each ε value. Uncertainties in the BICePs scores and derivatives come from the standard error of the mean across five independent scans along ε. (b) The derivative of the BICePs score (scatter dots) and the second derivative of the BICePs score (orange tangent lines) at each epsilon value. The dotted black line at $\varepsilon^* = 1.0$ shows the true value, which is where the derivative of the BICePs score equals zero. BICePs calculations are run using the Student's likelihood model for 100k steps with 8 replicas.

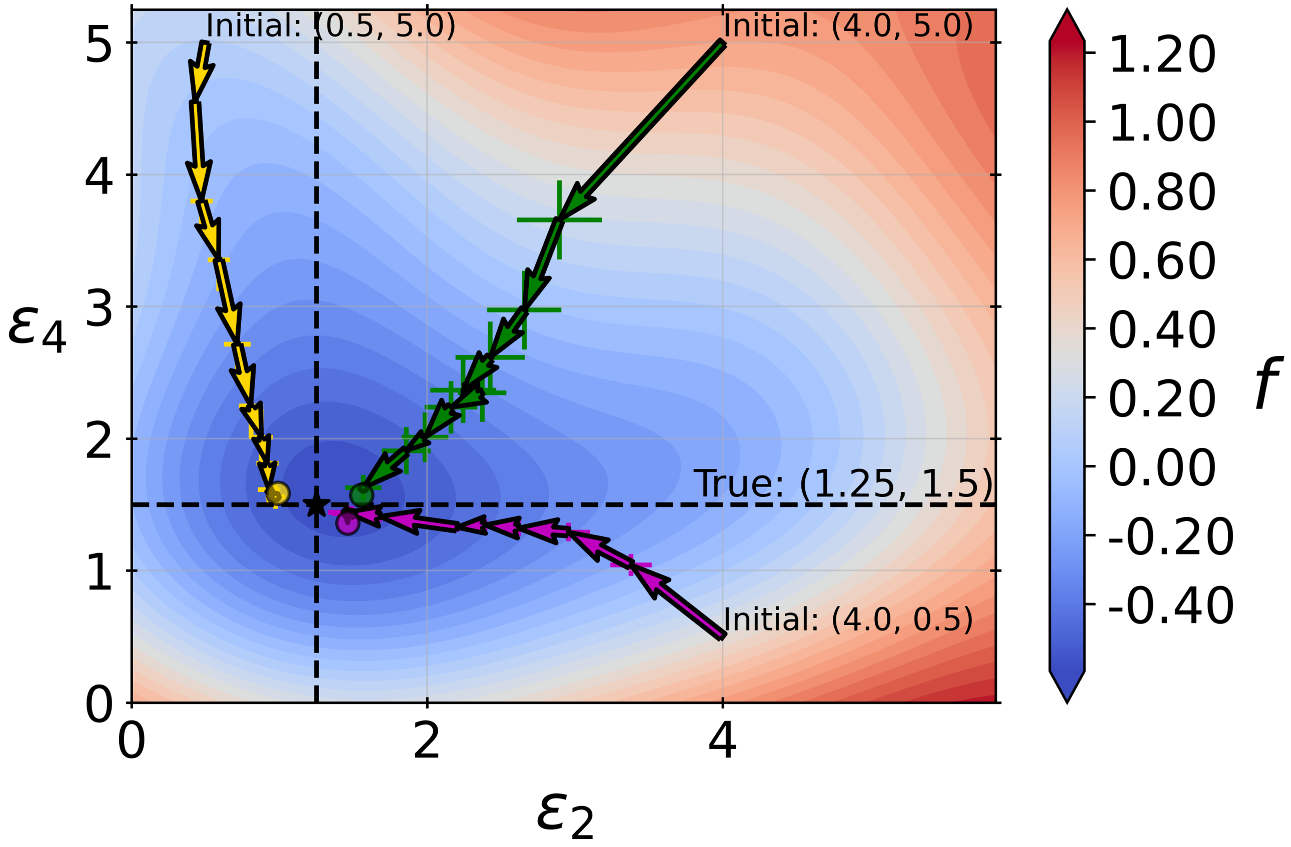

Figure S6. Average traces over a total of 25 independent rounds of parameter $(\varepsilon_2, \varepsilon_4)$ optimizations using first-order optimization method (L-BFGS-B) with BICePs, for a maximum of ten iterations. Traces are unable to fully reach the "True" iteration strength parameters ($\varepsilon_2^* = 1.25$, $\varepsilon_4^* = 1.5$) within 10 iterations when starting from different initial parameters $(\varepsilon_2^0, \varepsilon_4^0)$ = $\{(0.5, 5.0), (4.0, 5.0), (4.0, 0.5)\}$. The BICePs score landscape was generated from the average values of five scans over ε_2 and ε_4. All calculations used the Student's model with 200k MCMC steps and 8 replicas.

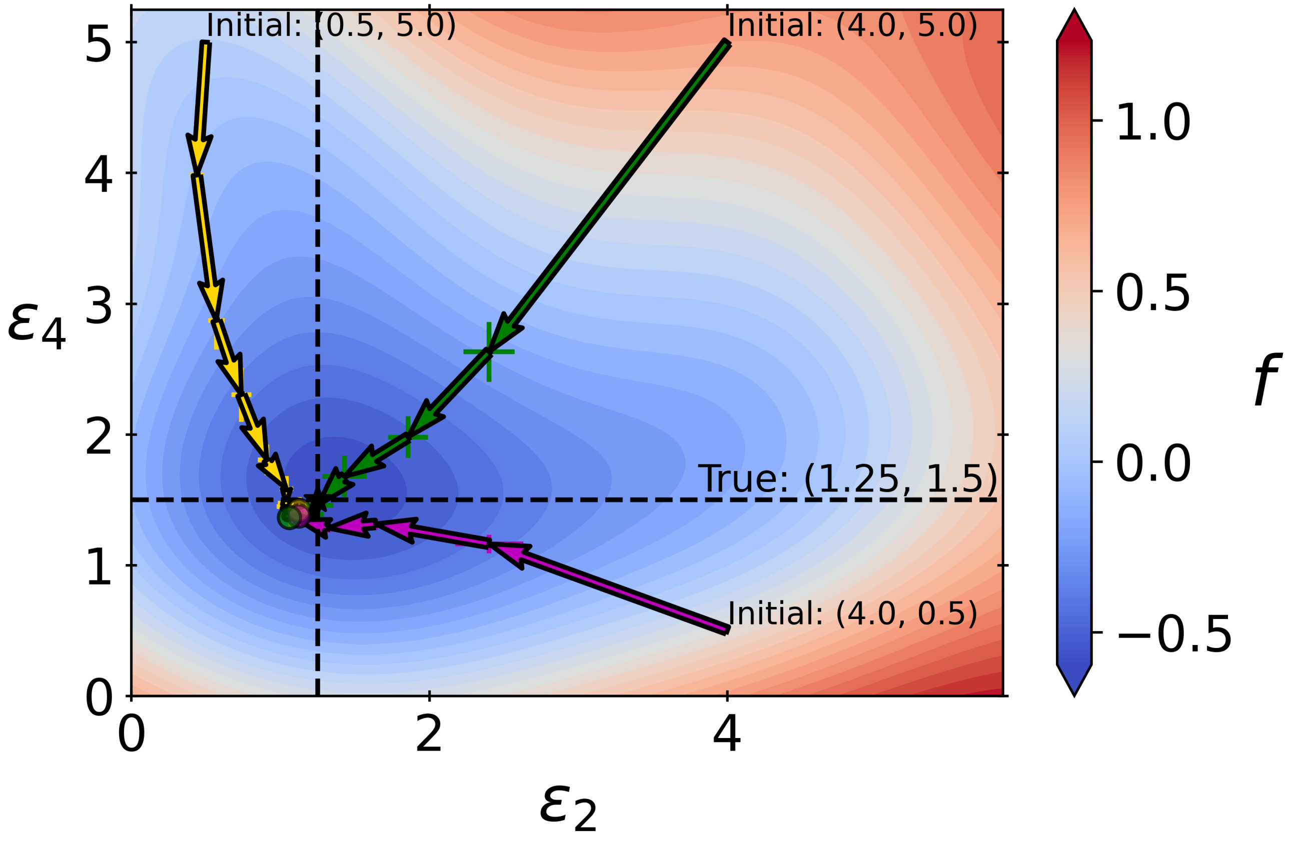

Figure S7. Average traces over a total of 25 independent rounds of parameter $(\varepsilon_2, \varepsilon_4)$ optimizations using second-order (trust-ncg) method with BICePs, for a maximum of ten iterations. Optimizations converge to the "True" optimal iteration strength parameters ($\varepsilon_2^* = 1.25$, $\varepsilon_4^* = 1.5$) when starting from different initial parameters $(\varepsilon_2^0, \varepsilon_4^0) = \{(0.5, 5.0), (4.0, 5.0), (4.0, 0.5)\}$. The BICePs score landscape was generated from the average values of five scans over ε_2 and ε_4. All calculations used the Student's model with 200k MCMC steps and 8 replicas. Average optimized parameter values were determined to be ($\varepsilon_2 = 1.07 \pm 0.85$, and $\varepsilon_4 = 1.46 \pm 0.82$), where the uncertainties are estimated from the inverse Hessian.

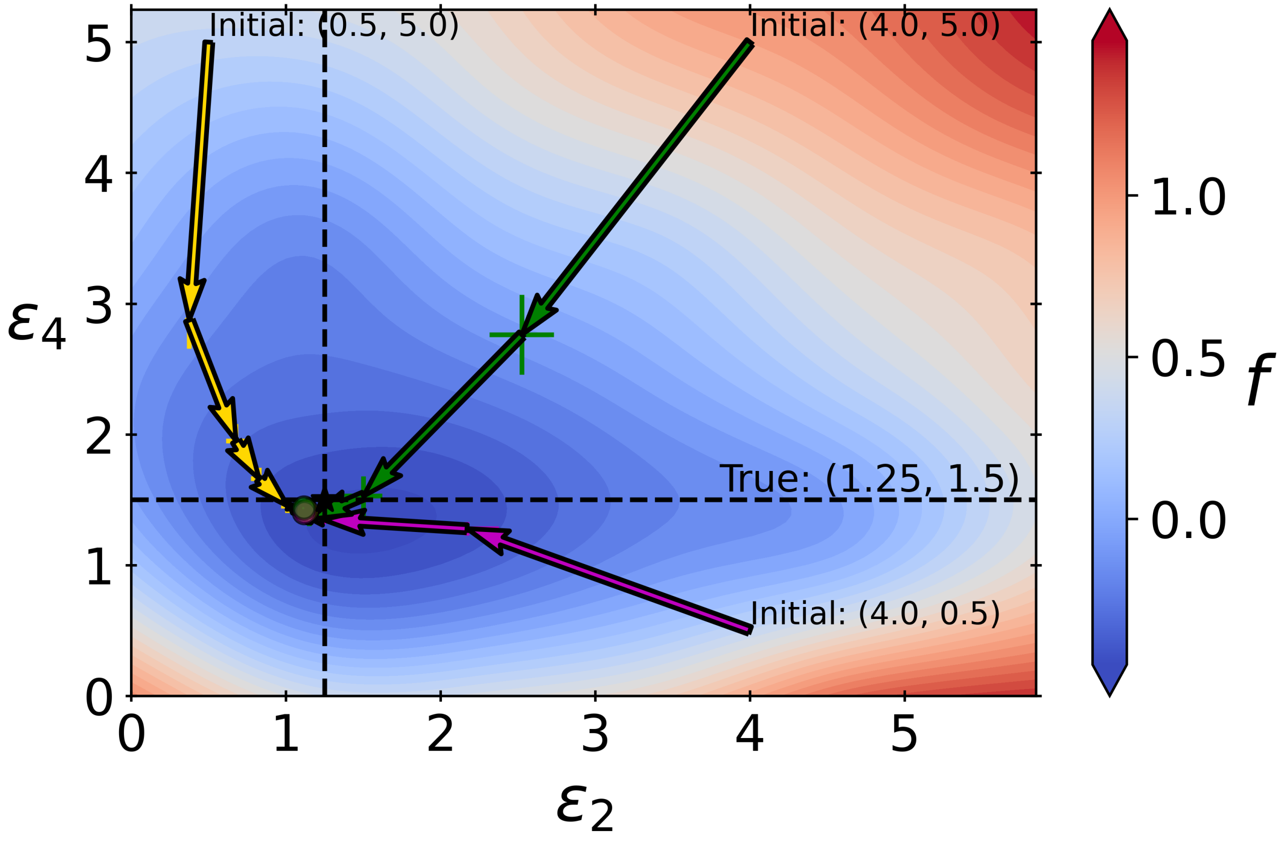

Figure S8. Average traces over a total of 25 independent rounds of parameter $(\varepsilon_2, \varepsilon_4)$ optimizations using second-order (trust-ncg) method with BICePs, for a maximum of ten iterations. In these tests, no error was added to the experimental data. Optimizations converge to the "True" parameters ($\varepsilon_2^* = 1.25$, $\varepsilon_4^* = 1.5$) when starting from different initial parameters $(\varepsilon_2^0, \varepsilon_4^0) = \{(0.5, 5.0), (4.0, 5.0), (4.0, 0.5)\}$. The BICePs score landscape was generated from the average values of five scans over ε_2 and ε_4. All calculations used the Student's model with 200k MCMC steps and 32 replicas. Average optimized parameter values were ($\varepsilon_2 = 1.12 \pm 0.42$, and $\varepsilon_4 = 1.43 \pm 0.36$), where the uncertainties are estimated from the inverse Hessian.

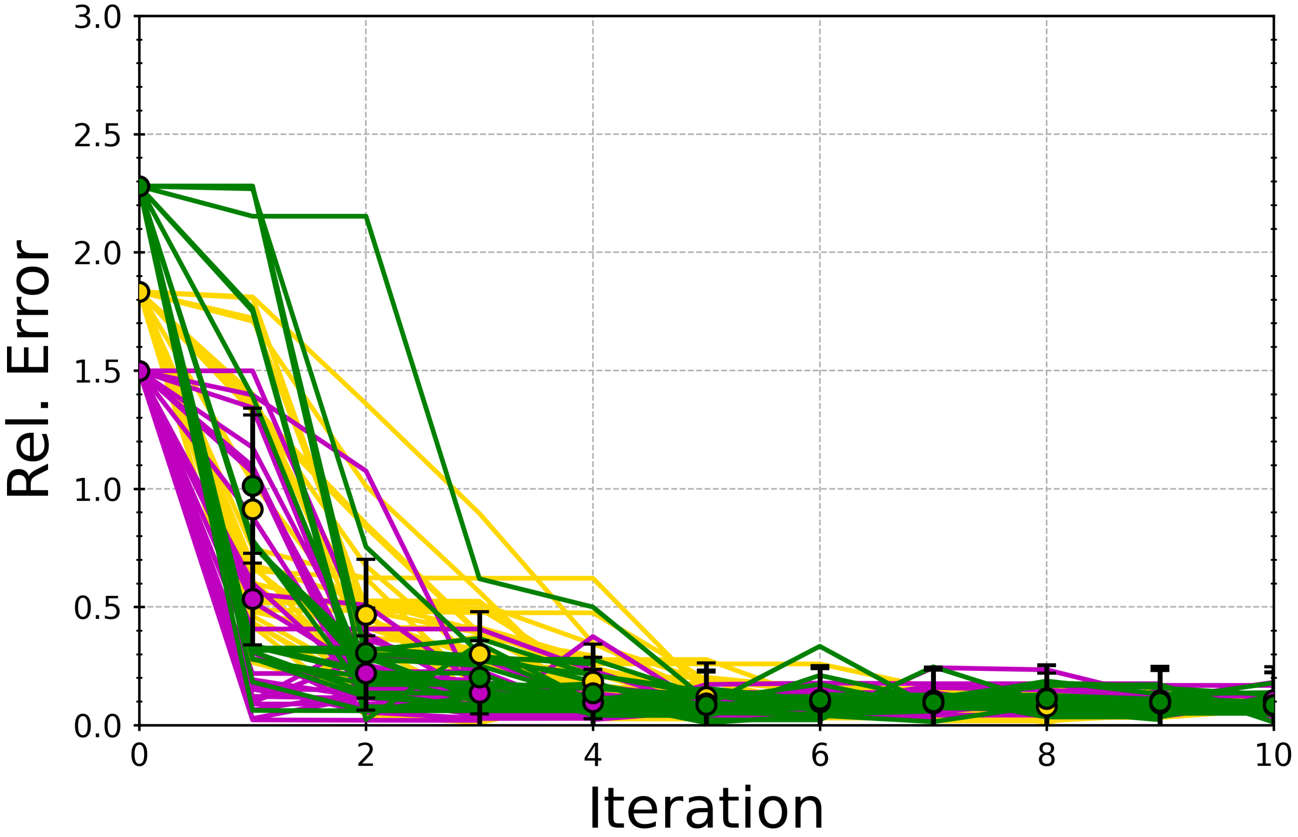

Figure S9. Accuracy profiles for $(\varepsilon_2, \varepsilon_4)$ optimizations using second-order (trust-ncg) method with BICePs, for a total of 25 independent rounds and a maximum of ten iterations. In these tests, no error was added to the experimental data. Optimizations converge to the "True" parameters ($\varepsilon_2^* = 1.25$, $\varepsilon_4^* = 1.5$) with a relative error less than 1.0 when starting from different initial parameters $(\varepsilon_2^0, \varepsilon_4^0)$ = $\{(0.5, 5.0), (4.0, 5.0), (4.0, 0.5)\}$. All calculations used the Student's model with 200k MCMC steps and 32 replicas. Average optimized parameter values were (ε_2 = 1.12 $\pm$ 0.42, and ε_4 = 1.43 $\pm$ 0.36), where the uncertainties are estimated from inverse Hessian. Error bars represent uncertainties from Monte Carlo error propagation using the σ_ε from the inverse Hessian, taken over all independent optimizations.

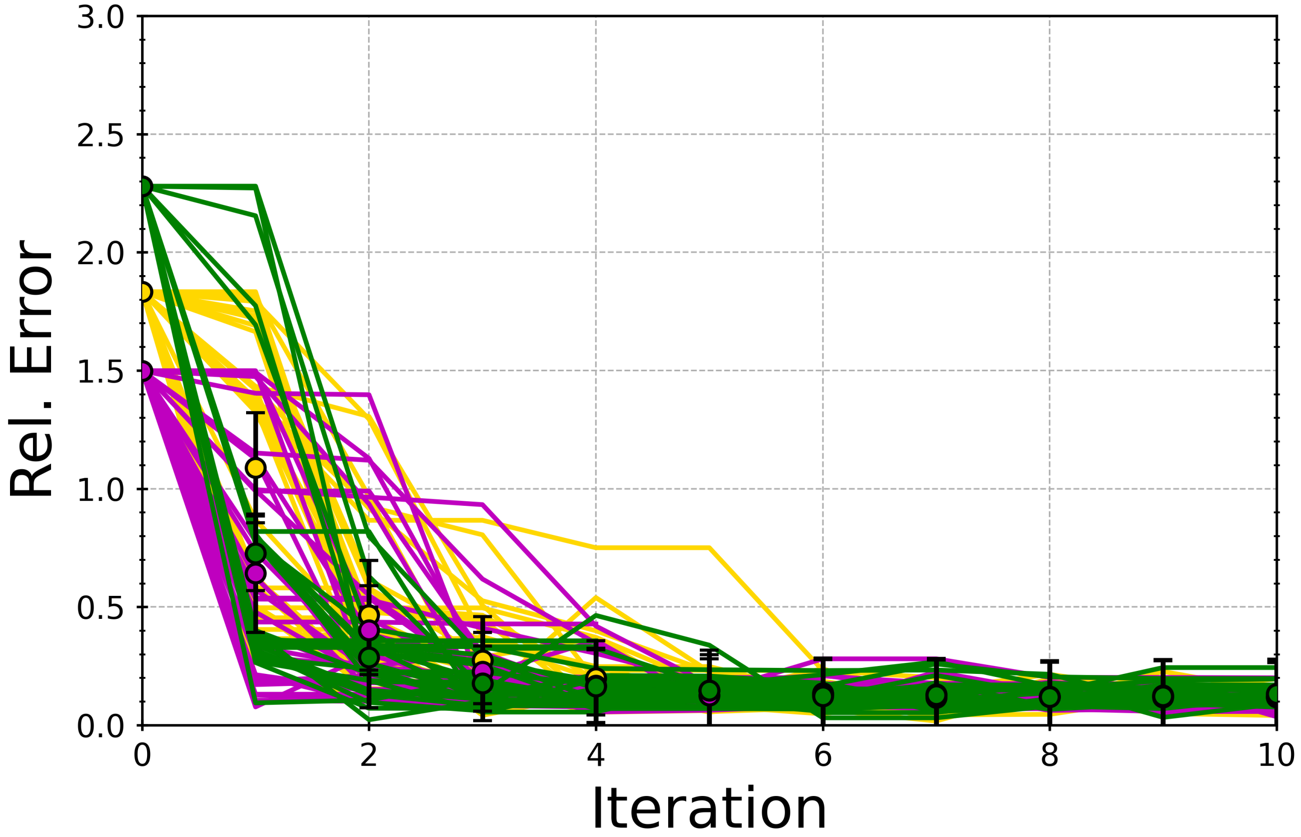

Figure S10. Accuracy profiles for $(\varepsilon_2, \varepsilon_4)$ optimizations using second-order (trust-ncg) method with BICePs, for a total of 25 independent rounds and a maximum of ten iterations. In these tests, the experimental data was corrupted with systematic error in the 2–11 and 4–9 distances for +3 and +3.5 L.U. shift, respectively. The total error in the data is $\sigma_{data} = 1.63$ L.U.. Optimizations converge to the "True" optimal iteration strength parameters ($\varepsilon_2^* = 1.25$, $\varepsilon_4^* = 1.5$) with a relative error less than 1.0 when starting from different initial parameters $(\varepsilon_2^0, \varepsilon_4^0)$ = $\{(0.5, 5.0), (4.0, 5.0), (4.0, 0.5)\}$. Error bars represent uncertainties estimated from the inverse Hessian, averaged over the 25 independent optimizations. All calculations used the Student's model with 200k MCMC steps and 32 replicas.

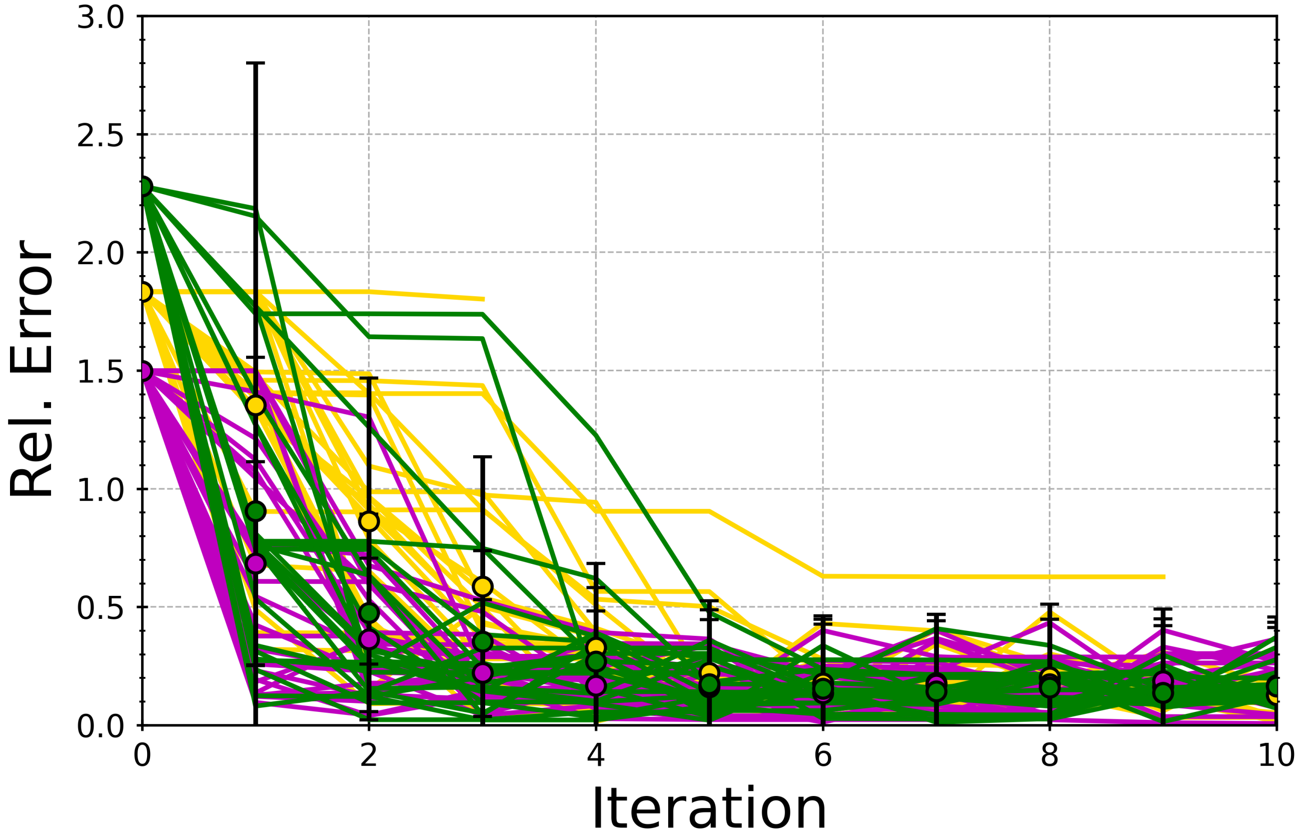

Figure S11. Accuracy profiles for $(\varepsilon_2, \varepsilon_4)$ optimizations using a second-order (trust-ncg) method with BICePs, for a total of 25 independent rounds and a maximum of ten iterations. In these tests, no error was added to the experimental data. Optimizations converge to the "True" parameters ($\varepsilon_2^* = 1.25$, $\varepsilon_4^* = 1.5$) with a relative error less than 1.0 when starting from different initial parameters $(\varepsilon_2^0, \varepsilon_4^0)$ = $\{(0.5, 5.0), (4.0, 5.0), (4.0, 0.5)\}$. Error bars represent uncertainties estimated from the inverse Hessian, averaged over the 25 independent optimizations. All calculations used the Student's model with 200k MCMC steps and 8 replicas. Average optimized parameter values were determined to be (ε_2 = 1.07 $\pm$ 0.85, and ε_4 = 1.46 $\pm$ 0.82), where the uncertainties are estimated from inverse Hessian. Error bars represent uncertainties from Monte Carlo error propagation using the σ_ε from the inverse Hessian, taken over all independent optimizations.

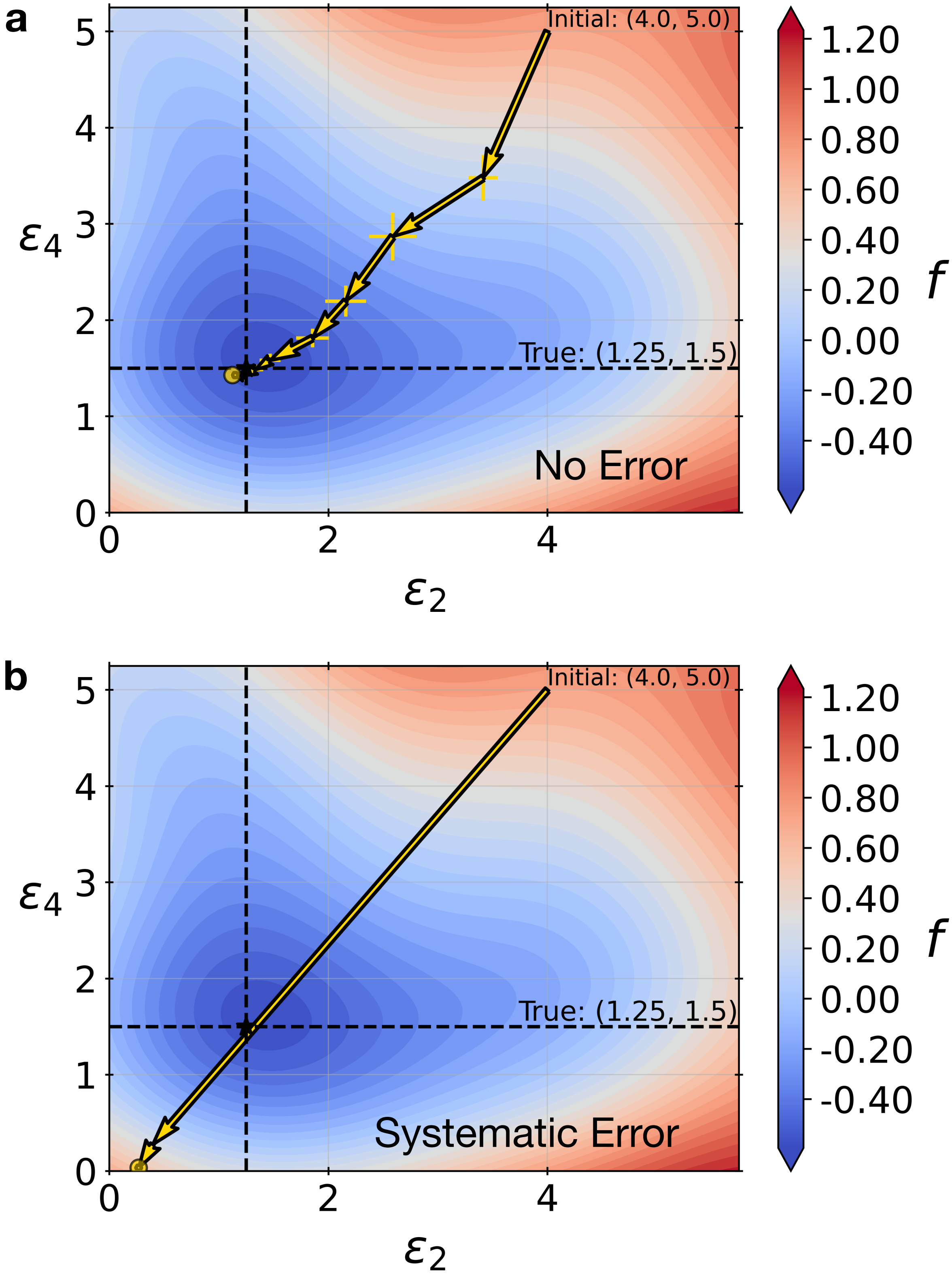

Figure S12. The accuracy of a standard Gaussian likelihood drops in the presence of systematic error. Demonstrating optimization accuracy when the data is corrupted with and without systematic error using $\varepsilon_2 = 4.0$ and $\varepsilon_4 = 5.0$ as initial parameters, same conditions as in Figure 5, where the "True" parameters are $(\varepsilon_2^* = 1.25, \varepsilon_4^* = 1.5)$. (a) The Gaussian likelihood performs great when using experimental data not affected by systematic error. (b) The accuracy suffers when the data is corrupted with systematic error in the 2-11 and 4-9 distances for +3 and +3.5 L.U. shift, respectively. The total error in the data is $\sigma_{data} = 1.63$. All results shown here use 8 replicas and are averages over 25 independent optimizations, and error bars are computed as standard error of the mean for each iteration.

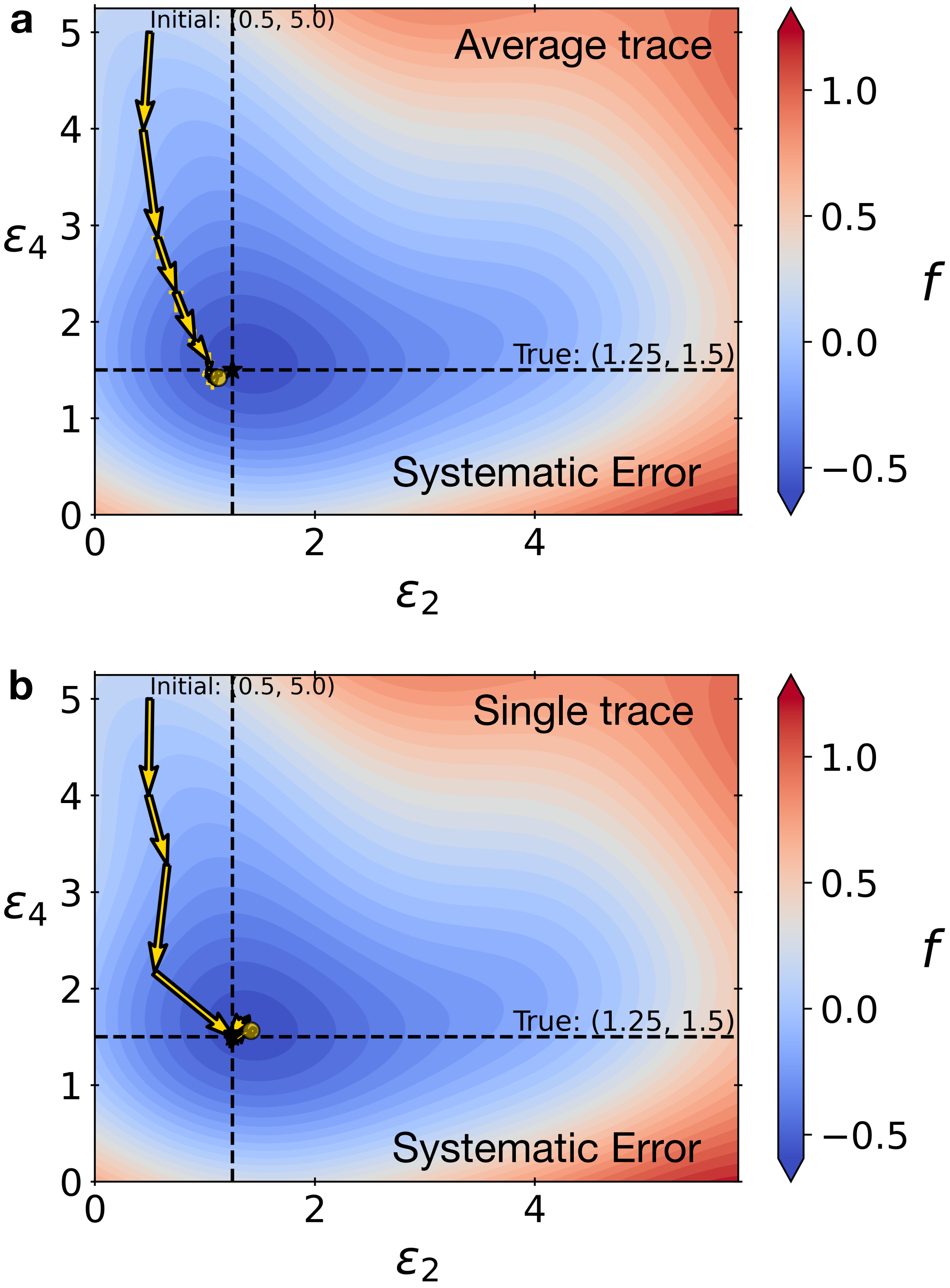

Figure S13. The accuracy of the Student's model is very robust in the presence of systematic error. (a) Average traces over 25 independent optimizations when the data is corrupted with systematic error in the 2–11 and 4–9 distances for +3 and +3.5 L.U. shift, respectively. The total error in the data is $\sigma_{data} = 1.63$. (b) A single representative trace demonstrating convergence to the "True" parameters ($\varepsilon_2^* = 1.25, \varepsilon_4^* = 1.5$). All results shown here use 8 replicas and are averages over 25 independent optimizations.

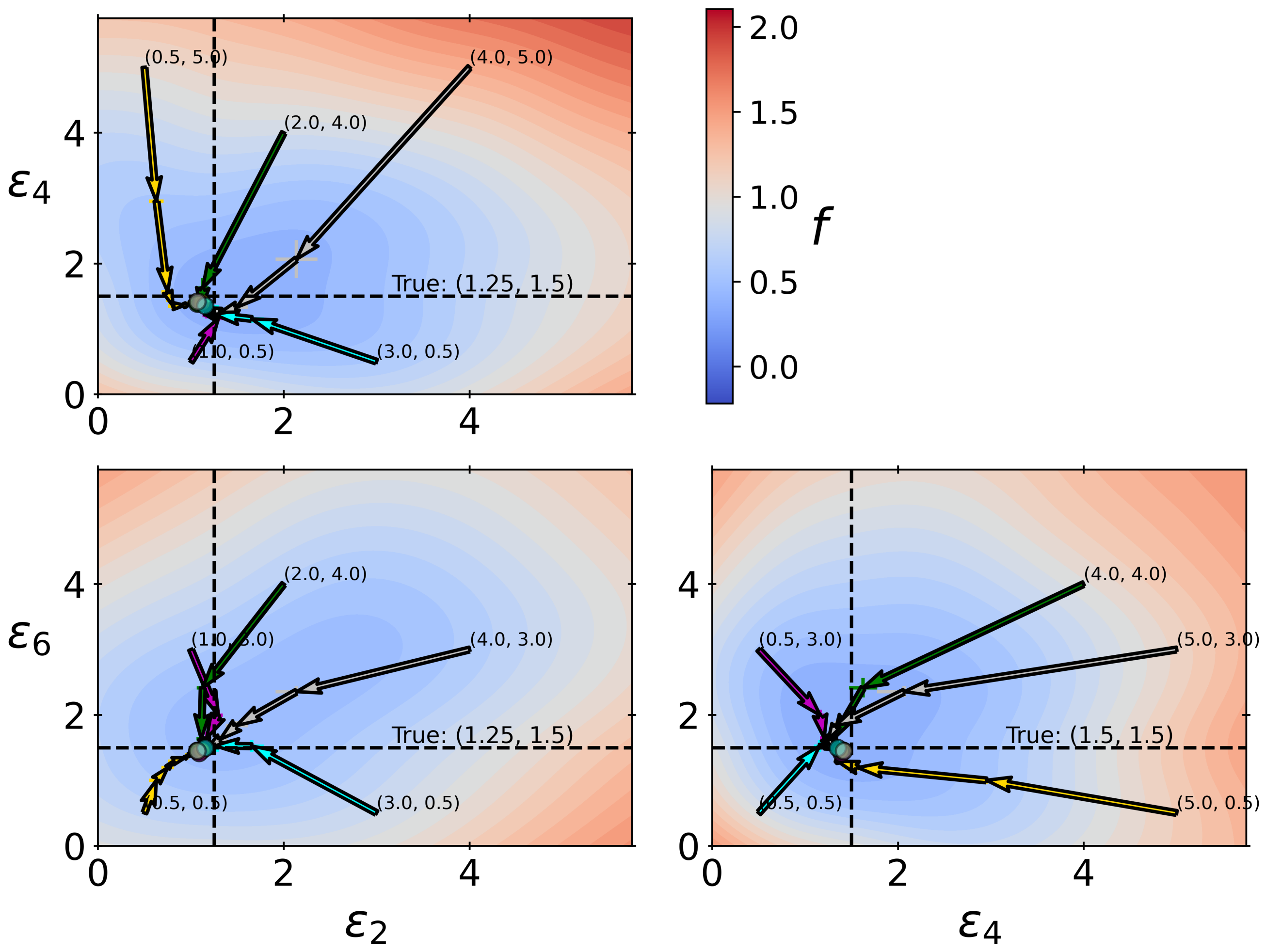

Figure S14. Average traces over a total of 25 independent rounds of parameter $(\varepsilon_2, \varepsilon_4, \varepsilon_6)$ optimizations using second-order (trust-ncg) method with BICePs, for a maximum of ten iterations. Optimizations converge to the "True" optimal iteration strength parameters ($\varepsilon_2^* = 1.25$, $\varepsilon_4^* = 1.5$, $\varepsilon_6^* = 1.5$) when starting from different initial parameters. All calculations used the Student's model with 200k MCMC steps and 32 replicas.

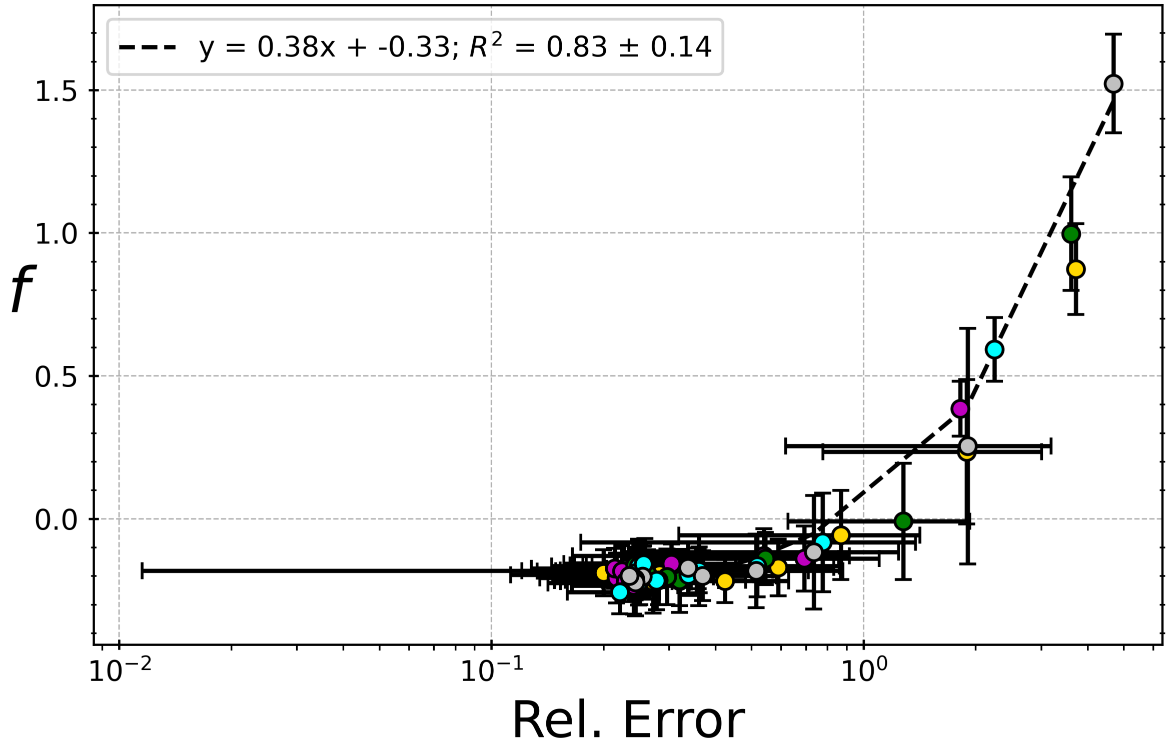

Figure S15. Sensitivity analysis of parameter $(\varepsilon_2, \varepsilon_4, \varepsilon_6)$ optimizations from various initial conditions. Regardless of different starting parameters, optimizations universally converge to "True" optimal iteration strength parameters ($\varepsilon_2^* = 1.25$, $\varepsilon_4^* = 1.5$, $\varepsilon_6^* = 1.5$) and succeed in diminishing the relative error. All BICePs score optimizations used 32 BICePs replicas with the Student's likelilhood model, and 200k MCMC steps per iteration. The dashed line is a linear fitting with equation $y = 0.38x - 0.33$, resulting in a strong correlation between the BICePs score and relative model error (coefficient of determination $R^2 = 0.83 \pm 0.14$).

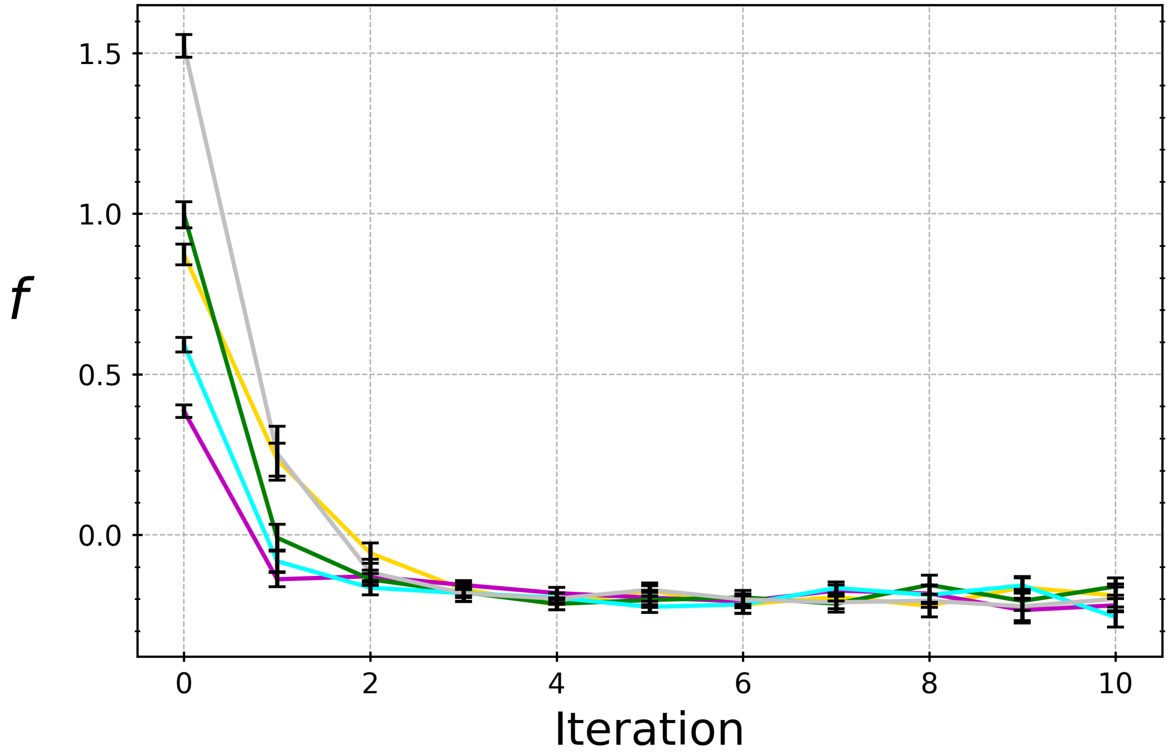

Figure S16. Minimization of the BICePs score, f is demonstrated when averaging over 25 independent rounds of parameter $(\varepsilon_2, \varepsilon_4, \varepsilon_6)$ optimizations using second-order (trust-ncg) method, for a maximum of ten iterations. Error bars represent the standard error over the 25 optimizations.

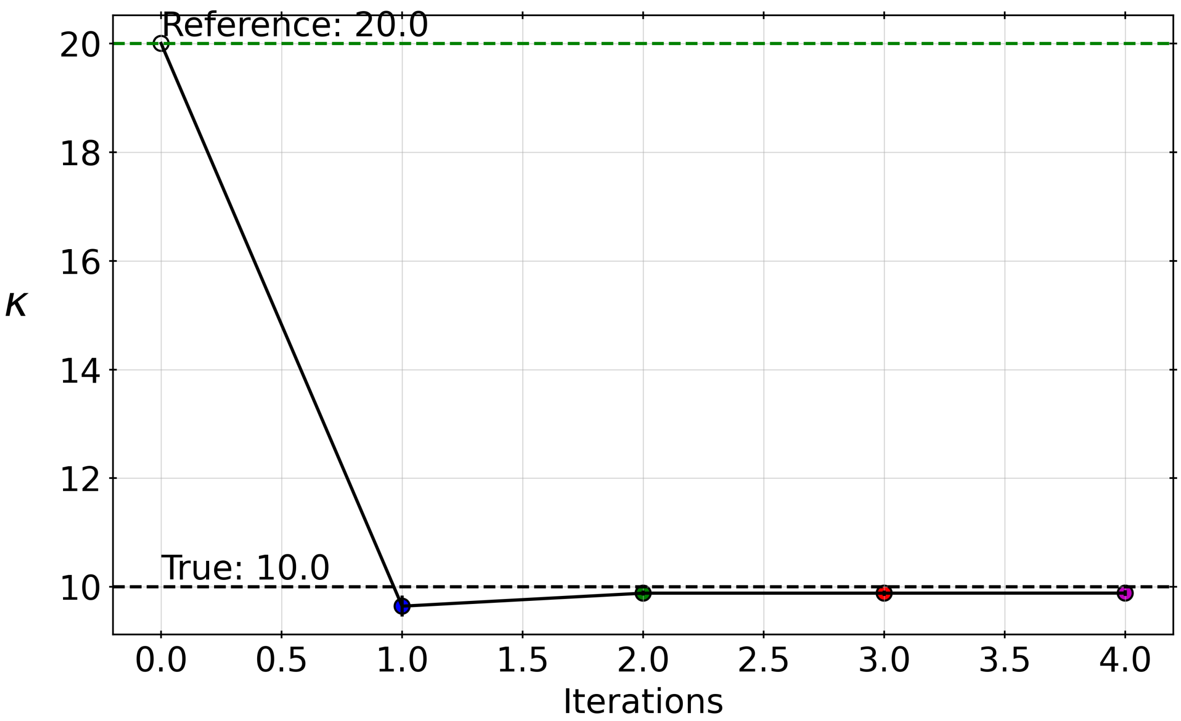

Figure S17. Refinement of the κ, the stiffness parameter in the KH polymer model. An initial parameter value, $\kappa^0 = 20$, was established. Employing second-order optimization via the 'trust-ncg' method, convergence to the "true" parameter, $\kappa^* = 10$, was achieved within a few iterations. The computations used 200 replicas and 100k MCMC steps, encompassing 500 configurations. Parameter uncertainties throughout each iteration were estimated from the inverse Hessian. These results successfully replicate the outcome of BioFF by Kofinger and Hummer.[7].

A

Number of Parameters	BICePs Sampling (s)	Jacobian (s)	Hessian (s)
2	2.75	0.55	0.93
3	2.80	1.15	2.50
4	2.82	1.56	4.26
5	2.84	1.99	6.55

B

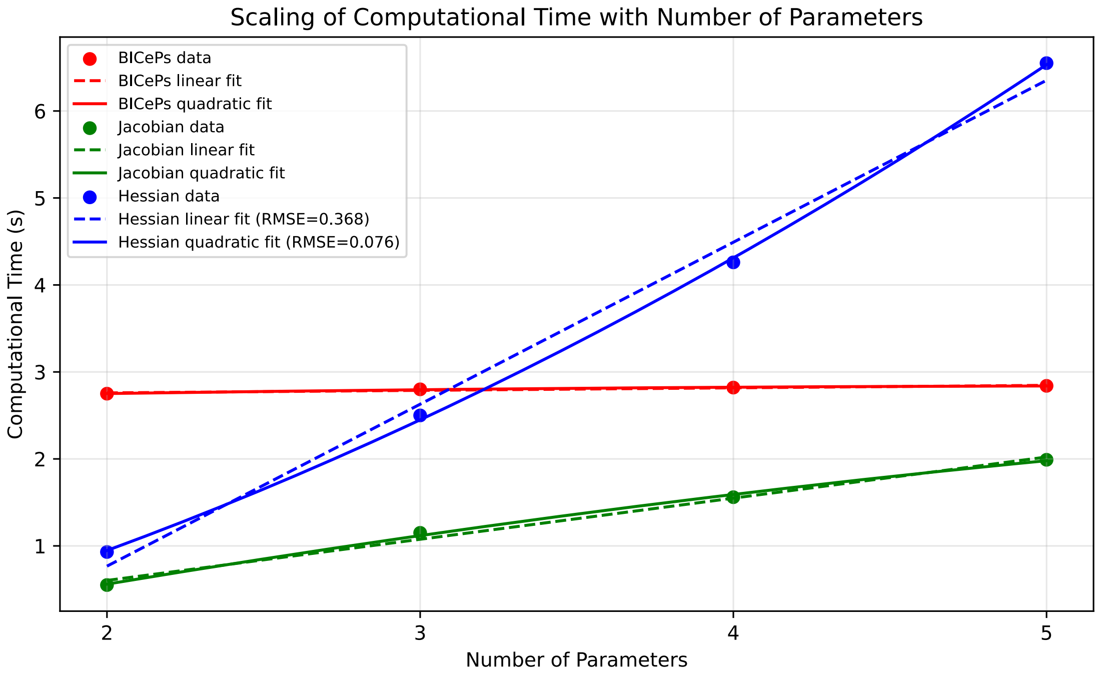

Figure S18. (A) Estimated computational runtimes for models with 2, 3, 4 and 5 parameters. All estimated runtimes were evaluated using a MacBook M1 Pro. (B) Linear and quadratic fits to the scaling data. For the Hessian scaling data, a quadratic curve is a visibly better fit (RMSE = 0.368) compared to a linear fit (RMSE = 0.076).
